# Effects of Acute High-Intensity Interval Exercise and High-Intensity Continuous Exercise on Inhibitory Function of Overweight and Obese Children

**DOI:** 10.3390/ijerph191610401

**Published:** 2022-08-21

**Authors:** Ligong Zhang, Dongshi Wang, Siwen Liu, Fei-Fei Ren, Lin Chi, Chun Xie

**Affiliations:** 1 China Wushu School, Beijing Sport University, Beijing 100084, China; 2 Faculty of Sports Science, Ningbo University, Ningbo 315211, China; 3 China Swimming College, Beijing Sport University, Beijing 100084, China; 4 Department of Physical Education, Beijing Language and Culture University, Beijing 100083, China; 5 School of Physical Education, Minnan Normal University, Zhangzhou 363000, China; 6 Department of Physical Education, Shanghai Jiao Tong University, Shanghai 200240, China

**Keywords:** high-intensity interval exercise, high-intensity continuous exercise, cognitive function, inhibitory function, overweight and obesity

## Abstract

This study aimed to examine whether a single bout each of high-intensity interval exercise (HIIE) and high-intensity continuous exercise (HICE) could improve inhibitory functions of overweight and obese children, and which mode of exercise was more beneficial. Seventy-two overweight and obese children, with (26.02 ± 1.05 kg/m^2^), aged 10–14 years (11.56 ± 1.03 years), were randomly assigned to three groups. The HIIE group completed a 30-min treadmill exercise session (5-min warm up, 20-min HIIE, and 5-min cool-down). The HICE group performed 30 min of rope skipping, while the control (CON) group watched a designated cartoon on a tablet computer for the same duration. Reaction time and number of errors in the Stroop test were determined before and after the intervention. The difference between pre- and post-test reaction time scores was higher in the HIIE and HICE groups than in the CON group, while the pre- and post-test difference in the number of errors was similar between groups. Overall, it is likely that both acute HIIE and HICE were similarly efficient in facilitating cognitive and inhibitory functions of children with overweight and obesity conditions, supporting the benefits of acute high-intensity exercise probability for cognitive functions of children in general, as well as of the population with overweight and obesity conditions.

## 1. Introduction

Overweight and obesity is a serious public health problem worldwide. A recent study published in Lancet Diabetes & Endocrinology showed that China has become the country with the largest overweight and obese population, with half of the adults and one-fifth of the children meeting the criteria for overweight or obesity [1]. According to Wang et al.’s prediction, the total number of overweight and obese children in China would reach 58.92 million by 2030, resulting in the enormous medical cost of 418 billion yuan, which would account for 22% of the total medical burden—a challenge that needs to be met urgently.

Inhibition is one of the core executive functions [2] that plays an extremely important role in people’s daily lives and mental health. Inhibition is essential for resisting bad habits, automatic behavior, and temptation and for accomplishing goals or adapting to conflict situations, as well as for maintaining a healthy weight [3]. However, a number of cross-sectional and meta-analytical studies have shown that children and adults with overweight and obesity exhibit impaired inhibitory function, which is reduced compared to that of their healthy-weight peers [3,4,5,6,7]. Moreover, childhood is a critical period for the development of inhibitory control, as childhood overweight and obesity often persists into adolescence and adulthood [8].

Robust and consistent evidence has shown that acute exercise can facilitate cognitive function [9,10,11], especially inhibitory function, in adults [12,13,14] and children [15,16]. A meta-analysis study, which contained 36 RCT studies, stated that acute exercise could significantly improve inhibitory control in healthy children and adolescents [17]. Interestingly, these beneficial effects have also been observed in overweight and obese populations [18,19,20]. However, numerous studies to date have focused on the long-term effects of exercise [20,21,22,23], with relatively few researches done on the influence of acute exercise on the inhibitory function of overweight and obese children [24].

There is increasing interest in the popular and emerging exercise intervention mode, high-intensity intermittent exercise (HIIE), which is proven to be more effective in improving inhibitory function than traditional moderate-intensity continuous exercise (MICE) [25,26], which is usually regarded as the most beneficial acute aerobic exercise intensity on inhibitory function. HIIE is usually defined as an exercise mode characterized by relatively short-term vigorous exercise (i.e., reaching a maximum heart rate greater than 85% in a short time), interspersed with short-term rest or low-intensity exercise for recovery [27]. Being highly efficient and time-saving, HIIE could also improve the body composition of obese individuals [28]. However, as previous studies have mostly focused on individuals with normal weight, it is unclear whether HIIE can effectively improve inhibition in obese people. To date, only a few studies have investigated this issue. Drigny, et al. [29] conducted an HIIE program with six obese subjects and found that inhibitory control (the Stroop task) was improved after the intervention. Quintero, et al. [30] examined whether HIIE has a positive effect on the inhibitory function of overweight male adults. However, the above studies had small sample sizes and lacked a control group. In addition, we conducted a preliminary study on the effects of acute HIIE on inhibitory control of 16 young male adults with obesity; the study had a between-subject design [24]. The results showed that the obese participants performed faster after acute HIIE compared to the control condition, suggesting that acute HIIE could improve reaction speed and inhibition efficiency. Moreover, a large body of studies has focused on the different effects of HIIE and MICE, but little is known about the differences between HIIE and high-intensity continuous exercise (HICE). Therefore, investigations of the effects of acute HIIE and HICE on inhibitory function of children with overweight and obesity are still at the preliminary stage, and larger sample sizes and more high-quality studies are warranted.

This study examined whether acute HIIE and HICE could improve inhibitory function of children with overweight and obesity, and further investigated which mode of exercise was more beneficial. We hypothesized that both exercise modes were effective in improving inhibitory control, and that HIIE could be a more effective regimen than HICE.

## 2. Materials and Methods

### 2.1. Participants

We recruited 72 children (more than 20 per group), aged 10–14 years, from the local area. Power calculation was performed by G*Power (G*Power 3.1 statistical software) [24,31,32]. Eligible participants met the following criteria: (a) overweight and obese status, with body mass index (BMI) meeting the International BMI criteria for overweight and obesity by sex of children between 2 and 18 years of age [33]; (b) no cardiovascular or related diseases; and (c) a healthy body that could withstand high-intensity exercise. 72 children were randomly assigned to three groups: the HIIE group (HIIE); the HICE group (HICE); and the control group (CON). The single sample ANOVA showed no significant difference among the three groups in relation to gender, age, height, weight, BMI, family income and educational level. The demographic data for the children are shown in Table 1. All participants and their guardians provided written informed consent in accordance with the Institutional Review Board at Shanghai University of Sport (#102772019RT005). 

### 2.2. The Stroop Task

The Stroop test [34] is a classical task, which is also called the Color Naming Task, and is usually used to assess cognitive function and the ability to inhibit habitual responses [35]. The test–retest reliability is 0.84 [36]. The effects of acute exercise on inhibition were derived from studies using the Stroop test in a great deal of the literature [11,13,37,38,39,40,41]. It included three conditions: the congruent; the neutral; and the incongruent. The stimuli in the congruent condition were color names painted in the same color (e.g., the word “blue” painted in blue), which is used to measure the processing speed (basic cognitive function). The stimuli in the incongruent condition were color names printed in different colors (e.g., the word “blue” painted in red), used to assess the inhibitory function (high-ordered cognitive function), specifically, the ability to make use of the cognitive control mechanism in order to inhibit word reading and activate the color-naming process [11,39]. The stimuli in the neutral condition were words unrelated to color in different colors (e.g., the word “teach” painted in purple). The stimuli of the Stroop test were displayed by using a paper format, and each of the conditions included 50 stimuli printed on a paper sheet. Participants were instructed to say each of the colors of the words as quickly as possible as they viewed the stimuli, and were well practiced before the formal test. When the subjects finished responding to all the stimuli, the well-trained examiner immediately pressed a hand-held stopwatch, then removed the stimuli paper, and recorded the subjects’ reaction times and whether their answers were correct or wrong. Then the examiner took over the stimuli paper and let participants answer the next stimuli. The number of errors and reaction times were recorded. 

### 2.3. Experimental Procedure

Participants were invited to the laboratory to first fill in a questionnaire, which asked for their age, family income, and years of education; the participants’ height and weight were also measured. Then, the HIIE group completed a single bout of 30-min treadmill exercise session, which included 5 min of 60–70% maximal heart rate (HR_max_ = 220 − age) [42] warm up, 8 × 2 min of 85–95% HR_max,_ interspersed with 1 min of relaxation (20 min in total), and 4 min of cool-down. The HICE group had a single bout of 30-min 80–85% HR_max_ rope skipping intervention. Every participant’s exercise regime was supervised by a fitness coach, so as to control and monitor his/her HR with a Polar (OH1, Polar Electro Malaysia (M) Sdn.Bhd) heart rate band. Once the heart rate of the subject was not within the target heart rate range, the coach would immediately remind him/her to speed up or slow down, in order to meet the exercise intensity range. The control group watched a designated cartoon on a tablet computer for 30 min. The Stroop test was conducted immediately before and after the intervention.

### 2.4. Statistical Analysis

The study used a mixed experimental design, with participant group as the between-subject factor and the stimulus condition as the within-subject factor. A 3 (group: HIIE vs. HICE vs. CON) × 3 (stimulus condition: congruent vs. incongruent vs. neutral) repeated-measures analysis of variance (ANOVA) was performed to analyze the pre-test and post-test difference (Δ) in reaction time and in the number of errors. Additionally, we conducted a 3 (group) × 3 (stimulus condition) × 2 (gender: male, female) repeated-measures analysis of variance (ANOVA), in order to examine whether gender could affect the behavioral results. The follow-up comparison adopted a Bonferroni adjustment, and the effect size is reported as (*η*^2^*_p_*), while the statistical significance was set at *p* < 0.05.

## 3. Results

### Behavior Data

**Figure 1:**
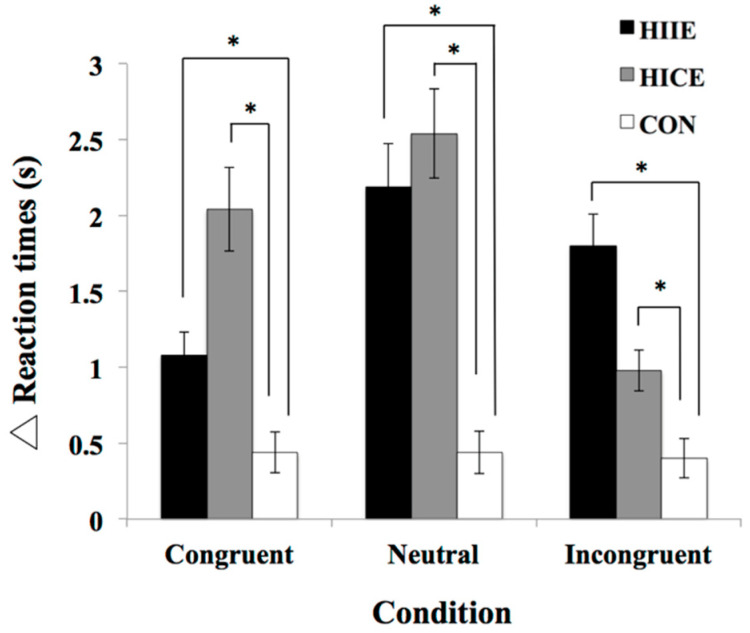
Mean and SE (standard error) of the Δ (difference between pre-test and post-test) reaction time in the Stroop task among the three groups. The High-intensity interval exercise (HIIE) group and the High-intensity continuous exercise (HICE) group showed larger Δ reaction time than the Control (CON) group in three stimulus conditions (Congruent, Neutral and Incongruent condition), whereas no significant difference was observed between the HIIE group and the HICE group. Note: * *p* < 0.001.

The 3 × 3 repeated-measures ANOVA of Δ reaction time showed a significant main effect of group (*F*_(2,69)_ = 13.71, *p* < 0.001, *η*^2^*_p_* = 0.28). The follow-up comparison revealed a significant difference between the HIIE group (1.69 ± 0.21 s) and the CON group (0.43 ± 0.21 s) as well as between the HICE group (1.85 ± 0.21 s) and the CON group, whereas no significant difference was observed between the HIIE group and the HICE group (*p =* 1.00). There was no main effect of stimulus condition (*F*_(2,138)_ = 3.01, *p* = 0.05, *η*^2^*_p_* = 0.04) or an effect of the interaction between participant group and stimulus condition (*F*_(4,138)_ = 2.35, *p* = 0.06, *η*^2^*_p_* = 0.06). The behavioral data for the Stroop task are presented in Table 2 and Figure 1.

**Figure 2:**
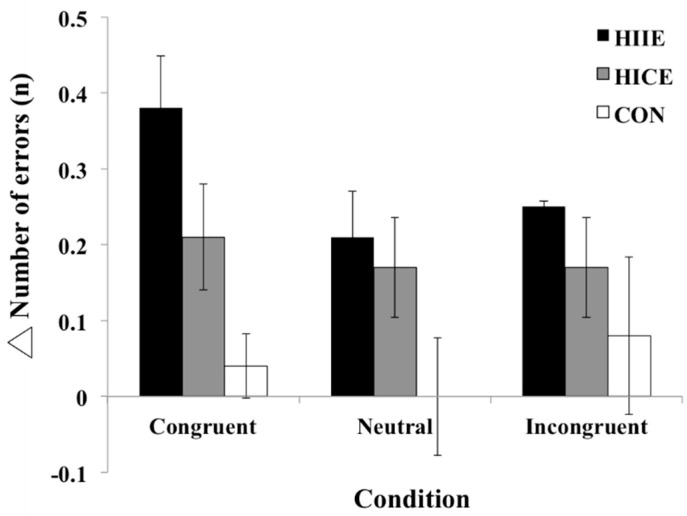
Mean and SE of the Δ (difference between pre-test and post-test) number of errors in the Stroop task among the three groups (The High-intensity interval exercise (HIIE) group, the High-intensity continuous exercise (HICE) group and the Control (CON) group). No significant difference and interactions among these three groups and three stimulus conditions (Congruent, Neutral and Incongruent condition) (*p* > 0.05).

The 3 × 3 repeated-measures ANOVA of Δ number of errors revealed no significant main effect of group (*F*_(2,69)_ = 2.92, *p* = 0.06, *η*^2^*_p_* = 0.08) or stimulus condition (*F*_(2,138)_ = 0.34, *p* = 0.72, *η*^2^*_p_* = 0.01) or interaction effect (*F*_(4,138)_ = 0.15, *p* = 0.96, *η*^2^*_p_* < 0.01). The behavioral data for the Stroop task are presented in Table 2 and Figure 2.

## 4. Discussion

This study examined the effects of popular exercise modes—acute HIIE and traditional acute HICE—on cognitive and inhibitory functions and compared the differences between the two exercise modalities. This is the first known study to investigate the influence of a single bout of HIIE and HICE on cognition of children with overweight and obesity. The results show that both acute HIIE and HICE could improve cognitive performance; however, there was no difference between the two exercise modes in this effect. Overall, the findings support the benefits of acute exercise for cognitive function in children and may predictably extend this effect to the population with overweight and obesity conditions.

Recent empirical studies [32,43,44,45] and systematic reviews [25,26] have focused on the effects of acute HIIE on healthy adults and children [46]. The results of our study further support the view that acute HIIE can enhance cognitive and inhibitory functions of adults with overweight and obesity [24,29,30] as well as children with obesity [46]. Our results also agree with earlier observations that showed shortened reaction times in inhibition tasks, suggesting improved speed and efficiency in cognitive function [24,32,43]. Another important finding was that the HICE group also showed enhancement in reaction time in the cognition test. This finding is consistent with the results of previous empirical studies [47,48] and a recent meta-analysis reporting an overall small effect of facilitation by high-intensity exercise compared to rest (*d* = 0.34) [48]. Overall, our findings indicated a facilitative effect of a single bout of HIIE or HICE on cognitive function in individuals with overweight and obesity.

Contrary to our expectations, this study did not find a significant difference between HIIE and HICE. A previous study found that Stroop test performance under HIIE was better than under HICE, reflecting that the inherent ecological practicability of HIIE is a better fit with the physical activity rhythm of children [46]. Given the different populations (healthy children vs. children with obesity) in the two studies and that Drollette et al. [16] found that individuals with lower inhibitory capacity may benefit the most from acute exercise, therefore it is possible that children with obesity could make greater improvements in cognitive tasks regardless of whether the exercise is high-intensity interval or continuous. Future studies should compare people with obesity and their normal-weight counterparts to confirm the differences between the populations.

It is somewhat surprising that no significant main effect was noted for the three stimulus conditions. A previous study found that the Stroop incongruent condition showed the largest positive effect after exercise intervention than four other conditions [49], indicating a selective benefit for high-order executive function [50]. A possible explanation for this may be that the difference score is relatively small in this study. Notably, an improvement in cognition was found regardless of whether the stimulus condition was neutral, congruent, or incongruent. These findings are in line with our earlier observations, which showed improvement not only in the congruent condition but also in the incongruent condition after an HIIE session [24]. Given that the Stroop task and Flanker task both reflect cognitive inhibition [51], the congruent condition indicates basic cognitive function and the incongruent condition represents high-order inhibitory control [4,52], suggesting that acute HIIE and HICE have a general facilitating effect on cognitive function.

The major strength of this study was that it included both genders and with a relatively large sample size, which was able to discern positive effects of acute HIIE and HICE on cognitive function of children with overweight and obesity. However, it also had some limitations. Firstly, this study is a pilot study and it only used the Stroop test to examine one of the core components of executive function; whether the positive effects of acute HIIE and HICE on inhibitory function could extend to other inhibitory tasks, such as the Flanker, Go/No go task and other components, such as shifting, updating [2], and planning [53] is still unclear. Caution is advised regarding broader generalization and future work is required to establish a more complete picture of the effects of acute HIIE and HICE on inhibitory function and executive function. Secondly, a within-subjects design or a randomized controlled trial (RCT) design study may be more rigorous, and it is warranted to replicate the findings. Thirdly, we only controlled and monitored the HR of the participants during the exercise intervention, in order to ensure that the participants reached the targeted heart rate range and, furthermore, future studies should record and report the heart rate of the subjects during the exercise, so as to make the results more clearly understandable and relevant. Besides, the brain mechanism underlying acute HIIE and cognitive function of children remains unclear and should be explored in future research. Lastly, there is abundant room to investigate the dose–response relationship between acute HIIE and cognitive function, for example the influence of exercise intensity, duration, and modality [26] as well as work recovery ratio [53], to further develop precise exercise prescriptions for individuals with overweight and obesity. 

## 5. Conclusions

Acute HIIE and HICE were very likely similarly efficient exercise modalities for facilitating cognitive function and inhibitory function of children with overweight and obesity conditions. These results support the benefits of acute high-intensity exercise for cognitive function in children and may predictably extend this effect to the population with overweight and obesity. Future studies should use RCT designs with larger sample sizes to examine the effects of HIIE and HICE on other subcomponents of cognitive function to elucidate the precise brain mechanisms underlying the effects.

## Figures and Tables

**Table 1:** Descriptions of the demographic data of the three groups.

Variable	HIIE Group (n = 24)	HICE Group (n = 24)	CON Group (n = 24)
Gender (Male)	19	19	19
Age (years)	11.58 ± 1.14	11.71 ± 1.16	11.38 ± 0.77
Height (m)	1.46 ± 0.06	1.45 ± 0.08	1.45 ± 0.05
Weight (kg)	55.24 ± 6.29	55.90 ± 8.32	54.21 ± 4.65
BMI (kg/m^2^)	25.88 ± 1.12	26.31 ± 1.21	25.88 ± 0.73
Family income (yuan)	42.68 ± 8.73	42.68 ± 10.06	45.51 ± 8.64
Educational level (years)	9.58 ± 1.14	9.71 ± 1.16	9.38 ± 0.77

Note: HIIE = High-intensity interval exercise; HICE = High-intensity continuous exercise; CON = Control.

**Table 2:** Mean and SD (standard deviation) of the pre-test, post-test and Δ of the Δ Stroop behavior data recorded among the three groups. Note: HIIE = High-intensity interval exercise; HICE = High-intensity continuous exercise; CON = Control; Δ = the difference between pre-test and post-test.

Variable		HIIE Group	HICE Group	CON Group
Congruent reaction time (s)	Pre-test	25.60 ± 7.06	24.33 ± 5.71	24.35 ± 5.13
	Post-test	24.52 ± 7.17	22.29 ± 5.95	23.91 ± 4.97
	Δ	1.08 ± 1.27	2.04 ± 2.33	0.44 ± 1.14
Neutral reaction time (s)	Pre-test	33.53 ± 11.51	30.50 ± 8.62	32.33 ± 8.08
	Post-test	31.34 ± 10.37	27.96 ± 7.61	31.89 ± 7.44
	Δ	2.19 ± 2.39	2.54 ± 2.50	0.44 ± 1.19
Incongruent reaction time (s)	Pre-test	40.58 ± 9.05	40.17 ± 7.63	39.57 ± 6.03
	Post-test	38.78 ± 8.68	39.18 ± 7.71	39.17 ± 5.70
	Δ	1.80 ± 1.76	0.98 ± 1.15	0.40 ± 1.09
Congruent number of errors (n)	Pre-test	1.04 ± 0.91	0.79 ± 0.78	0.79 ± 0.78
	Post-test	0.67 ± 0.72	0.58 ± 0.78	0.75 ± 0.68
	Δ	0.38 ± 0.58	0.21 ± 0.59	0.04 ± 0.36
Neutral number of errors (n)	Pre-test	0.04 ± 0.36	0.83 ± 0.92	0.87 ± 1.03
	Post-test	0.96 ± 0.86	0.67 ± 0.76	0.88 ± 0.68
	Δ	0.21 ± 0.51	0.17 ± 0.56	0.00 ± 0.66
Incongruent number of errors (n)	Pre-test	1.13 ± 1.04	1.04 ± 0.91	1.21 ± 0.88
	Post-test	0.88 ± 0.74	0.88 ± 0.68	1.13 ± 0.85
	Δ	0.25 ± 0.61	0.17 ± 0.56	0.08 ± 0.88

## Data Availability

The data presented in this study are available on request from the corresponding author.
